# Occupational risk variation of nasopharyngeal cancer in the Nordic countries

**DOI:** 10.1186/s12885-022-10209-y

**Published:** 2022-11-04

**Authors:** Timo Carpén, Evelina Gille, Lalle Hammarstedt-Nordenvall, Johnni Hansen, Sanna Heikkinen, Elsebeth Lynge, Jenny Selander, Ingrid Sivesind Mehlum, Jóhanna Eyrún Torfadottir, Antti Mäkitie, Eero Pukkala

**Affiliations:** 1 Department of Otorhinolaryngology – Head and Neck Surgery, University of Helsinki and Helsinki University Hospital, FI-00029 HUS, Helsinki, Finland; 2 Department of Pathology, University of Helsinki and Helsinki University Hospital, FI-00014 Helsinki, Finland; 3 Research Program in Systems Oncology, Faculty of Medicine, FI-00014 University of Helsinki, Helsinki, Finland; 4 Division of Ear, Nose and Throat Diseases, Department of Clinical Sciences, Intervention and Technology, Karolinska Institutet and Karolinska Hospital, SE-17176 Stockholm, Sweden; 5 Danish Cancer Society Research Center, DK-2100 Copenhagen, Denmark; 6 Finnish Cancer Registry, Institute for Statistical and Epidemiological Cancer Research, FI-00130 Helsinki, Finland; 7 Nykøbing Falster Hospital, University of Copenhagen, DK-4800 Nykøbing Falster, Denmark; 8 Institute of Environmental Medicine – IMM Karolinska Institutet, S-17177 Stockholm, Sweden; 9 National Institute of Occupational Health (STAMI), N- 0363 Oslo, Norway; 10 Institute of Health and Society, University of Oslo, N-0450 Oslo, Norway; 11 Icelandic Cancer Registry, IS-105 Reykjavik, Iceland; 12 Centre of Public Health Sciences, Faculty of Medicine, University of Iceland, IS-102 Reykjavik, Iceland; 13 Health Sciences Unit, Faculty of Social Sciences, FI-33014 Tampere University, Tampere, Finland

**Keywords:** Incidence, Cohort, Nasopharyngeal cancer, Occupation, Risk factor

## Abstract

**Background:**

The aim of this study was to estimate occupational risk variation in the incidence of nasopharyngeal cancer (NPC) in a large population-based cohort of the Nordic Occupational Cancer (NOCCA) study.

**Methods:**

This study is based on a cohort of almost 15 million persons from Denmark, Finland, Iceland, Norway and Sweden, with 2898 nasopharyngeal cancer cases diagnosed in 1961–2005. The data on occupations were gathered from population censuses and cancer data from the national cancer registries. Standardized incidence ratios (SIR) with 95% confidence intervals (CI) were estimated using the national NPC incidence rates as the reference.

**Results:**

There were 1980 male and 918 female NPC patients. The highest SIRs of NPC were observed among male waiters (SIR 3.69, 95% CI 1.91–6.45) and cooks and stewards (SIR 2.24, 95% CI 1.16–3.91). Among women, launderers had the highest SIR of NPC (2.04, 95% CI 1.02–3.65). Significantly decreased SIRs were found among male farmers (SIR 0.79, 95% CI 0.68–0.92) and male textile workers (SIR 0.49, 95% CI 0.22–0.93).

**Conclusions:**

This study suggests that NPC may be associated with several work-related exposure agents such as smoking, kitchen air pollution and solvents. In future, occupational exposure-risk relations should be studied to understand more about causality and to assess effective prevention strategies.

## Background

Close to 850,000 new head and neck squamous cell carcinoma (HNSCC) cases are diagnosed annually worldwide, and approximately 130,000 of these are nasopharyngeal carcinomas (NPC) [1]. The NPC incidence is much higher in South-Asia than in Europe and North America [1, 2]. The majority of NPC cases in endemic areas are non-keratinizing and predominantly associated with the Epstein-Barr virus (EBV) [3]. Another major risk factor for NPC is tobacco smoking [4–6].

Exposure to occupation-related agents such as formaldehyde and chlorophenol, wood and cotton dust, smokes, and combustion have been associated with the development of NPC [7–12]. Upper airways including the nasopharynx are directly affected by these often work-related exposures. For example, occupational exposures to formaldehyde and wood dust have been classified by the International Agency for Research on Cancer (IARC) as having sufficient evidence of causing NPC in humans [13]. Long-lasting wood dust exposure especially appears to increase the risk of NPC [14–19]. Cotton dust, which is common in the textile industry, is another exposure that has been linked with NPC, but evidence remains inconclusive [20, 21].

The aim of this study was to identify occupations linked to NPC in the five Nordic countries.

## Methods

This study is based on NPC patients included in the Nordic Occupational Cancer (NOCCA) study cohort. The NOCCA cohort includes census participants covering a total of 14.9 million persons from Denmark, Finland, Iceland, Norway and Sweden followed up between 1961 and 2005 [22].

Subjects aged 30–64 years old and alive on January 1 of the year after the first available census were linked to their occupational data and cancer data with the help of the unique personal identity codes given to all residents of the Nordic countries [23]. The follow-up time of each individual started from the year following the first available census record of the person and ended at the date of emigration, death or at the end of the following years: Finland and Sweden 2005, Iceland 2004 and Norway and Denmark 2003 [24]. The present study used the occupational data that were originally coded according to Nordic Occupational Classification (NYK), International Standard of Classification of Occupations (ISCO) versions 58 and 68, or a special Danish classification were classified into 54 occupational categories. A detailed description of the NOCCA cohort and occupational data are presented in previous reports [23, 25]. For this study, all health care occupations were combined as the group of “health workers” because the number of cases in each category was low. Only occupational categories with ≥5 cases (Nordic total) are shown separately in the tables. The categories with less than 5 cases were added into the category “other economically active”.

Data on NPCs (International Classification of Diseases, 7th Revision, code 146) were obtained from the cancer registry of each country. During the follow-up period, all cancer registries received clinical notifications from hospitals and all except Denmark also from cancer laboratories in the public and private sectors [26].

The standardized incidence ratio (SIR) was used as the measure of relative risk of NPC. SIR is the ratio of the observed and expected numbers of cases. The expected number of cases in each country, gender, 5-year calendar period and 5-year age category was calculated by multiplying the number of person-years in the stratum by the respective national incidence rate of NPC. Exact 95% confidence intervals (CI) were determined by Poisson distribution, and the SIR was considered as statistically significant if its CI did not include 1.0. The study has permissions as required in each of the Nordic countries.

## Results

Altogether, 2898 patients diagnosed with NPC were observed during the follow-up of the present study. There were 1980 male and 918 female NPC patients. The majority (*n* = 1524) of the patients were from Sweden, and only 18 patients were from Iceland.

The highest SIR of NPC was found among male waiters in the whole Nordic cohort (SIR 3.69, 95% CI 1.91–6.45) and separately in Norway (SIR 4.79, 95% CI 1.30–12.25) and Denmark (SIR 6.81, 95% CI 2.21–15.88) (Table 1). The SIR for female waiters was not significantly elevated (Table 2).

**Table 1:** Observed (Obs) and expected (Exp) numbers of cancer of the nasopharynx and standardised incidence ratios (SIR) among men, by country and occupation. Only occupational categories with ≥5 cases (Nordic total) are shown. Country-specific numbers for Iceland not shown

Occupational category	Denmark				Finland				Norway				Sweden				Nordic total			
	Obs	Exp	SIR	95% CI	Obs	Exp	SIR	95% CI	Obs	Exp	SIR	95% CI	Obs	Exp	SIR	95% CI	Obs	Exp	SIR	95% CI
Technical workers. Etc	21	20.7	1.01	0.63–1.55	21	17.1	1.23	0.76–1.87	5	13.6	**0.37**	0.12–0.86	83	90.2	0.92	0.73–1.14	130	141.9	0.92	0.77–1.09
Health workers	0	4.1	**0.00**	0.00–0.97	4	2.3	1.76	0.48–4.51	5	3.6	1.39	0.45–3.24	10	12.1	0.83	0.40–1.52	19	22.2	0.86	0.51–1.34
Teachers	5	9.1	0.55	0.18–1.29	4	6.7	0.60	0.16–1.53	10	9.8	1.02	0.49–1.87	15	23.4	0.64	0.36–1.06	36	49.3	0.73	0.51–1.01
Artistic workers	1	1.2	0.87	0.02–4.84	1	1.3	0.74	0.02–4.14	2	1.5	1.37	0.17–4.95	4	5.63	0.71	0.19–1.82	8	9.7	0.83	0.36–1.63
Religious workers etc	1	3.6	0.28	0.01–1.56	2	4.1	0.49	0.06–1.76	3	4.9	0.61	0.13–1.78	18	19.6	0.92	0.54–1.45	25	32.5	0.77	0.50–1.14
Administrators	15	22.2	0.68	0.38–1.12	18	10.0	**1.79**	1.06–2.83	12	17.3	0.69	0.36–1.21	36	34.4	1.05	0.73–1.45	81	84.3	0.96	0.76–1.19
Clerical workers	15	10.1	1.48	0.83–2.44	4	6.4	0.63	0.17–1.61	12	13.2	0.91	0.47–1.59	30	37.4	0.80	0.54–1.14	61	67.7	0.90	0.69–1.16
Sales agents	5	5.4	0.93	0.30–2.16	17	9.5	**1.79**	1.04–2.86	9	15.3	0.59	0.27–1.11	51	55.2	0.92	0.69–1.21	83	85.8	0.97	0.77–1.20
Shop workers	27	22.3	1.21	0.80–1.76	7	4.3	1.63	0.66–3.36	6	6.1	0.98	0.36–2.14	20	17.3	1.15	0.71–1.78	60	50.3	1.19	0.91–1.54
Farmers	26	40.1	**0.65**	0.42–0.95	22	38.1	**0.58**	0.36–0.88	37	40.5	0.91	0.64–1.26	80	89.4	0.89	0.71–1.11	165	208.8	**0.79**	0.68–0.92
Gardeners	4	5.8	0.69	0.19–1.76	10	6.1	1.64	0.79–3.02	5	10.2	0.49	0.16–1.15	37	33.6	1.10	0.78–1.52	56	55.7	1.00	0.76–1.31
Fishermen	5	2.2	2.32	0.75–5.42	1	0.5	1.94	0.05–10.79	22	13.4	**1.64**	1.03–2.49	1	3.6	0.28	0.01–1.55	29	20.4	1.42	0.95–2.04
Forestry workers	0	0.8	0.00	0.00–4.38	9	7.4	1.22	0.56–2.31	4	7.5	0.53	0.15–1.36	15	24.7	0.61	0.34–1.00	28	40.4	0.69	0.46–1.00
Miners and quarry workers	1	0.3	3.69	0.09–20.54	4	1.2	3.27	0.89–8.38	4	2.2	1.79	0.49–4.59	3	6.3	0.47	0.10–1.38	12	10.1	1.19	0.62–2.08
Seamen	3	2.5	1.22	0.25–3.57	3	1.6	1.84	0.38–5.38	18	11.4	1.58	0.93–2.49	7	5.6	1.26	0.50–2.59	31	21.2	1.46	0.99–2.07
Transport workers	3	4.9	0.62	0.13–1.80	4	4.6	0.87	0.24–2.23	7	5.6	1.25	0.50–2.58	18	17.9	1.01	0.60–1.59	32	33.2	0.96	0.66–1.36
Drivers	23	16.7	1.38	0.87–2.07	18	15.7	1.14	0.68–1.81	16	16.4	0.97	0.56–1.58	54	46.1	1.17	0.88–1.53	113	95.4	1.19	0.99–1.42
Postal workers	4	3.3	1.22	0.33–3.13	3	2.4	1.27	0.26–3.71	4	3.1	1.29	0.35–3.29	14	10.3	1.36	0.75–2.29	25	19.1	1.31	0.85–1.94
Textile workers	1	2.8	0.36	0.01–2.01	0	1.4	0.00	0.00–2.61	0	3.3	0.00	0.00–1.13	8	10.9	0.74	0.32–1.45	9	18.4	**0.49**	0.22–0.93
Shoe and leather workers	0	0.7	0.00	0.00–5.55	0	0.6	0.00	0.00–6.04	2	1.4	1.42	0.17–5.12	7	4.36	1.61	0.65–3.31	9	7.06	1.27	0.58–2.42
Smelting workers	6	6.7	0.89	0.33–1.94	0	2.3	0.00	0.00–1.63	1	4.6	0.22	0.01–1.21	21	17.6	1.19	0.74–1.82	28	31.3	0.89	0.59–1.29
Mechanics	15	17.7	0.85	0.47–1.40	10	15.9	0.63	0.30–1.15	27	22.6	1.19	0.79–1.74	82	82.8	0.99	0.79–1.23	135	139.5	0.97	0.82–1.15
Plumbers	1	1.6	0.61	0.02–3.42	0	2.7	0.00	0.00–1.37	2	2.7	0.75	0.09–2.70	11	9.5	1.15	0.58–2.07	14	16.6	0.84	0.46–1.41
Welders	Undetermined				2	2.8	0.71	0.09–2.58	5	3.0	1.65	0.54–3.86	12	10.9	1.10	0.57–1.92	19	16.8	1.13	0.68–1.77
Electrical workers	4	4.4	0.92	0.25–2.35	6	7.1	0.85	0.31–1.85	10	10.0	1.00	0.48–1.85	27	28.8	0.94	0.62–1.36	47	50.5	0.93	0.68–1.24
Wood workers	4	11.8	**0.34**	0.09–0.87	15	15.3	0.98	0.55–1.62	26	25.0	1.04	0.68–1.53	70	54.6	1.28	1.00–1.62	115	106.9	1.08	0.90–1.29
Painters	5	4.0	1.25	0.41–2.93	4	3.3	1.19	0.33–3.06	3	4.3	0.69	0.14–2.02	15	16.0	0.94	0.52–1.54	27	27.8	0.97	0.64–1.41
Bricklayers	11	4.4	**2.53**	1.26–4.52	3	1.6	1.86	0.38–5.45	6	2.5	2.36	0.87–5.14	8	7.1	1.13	0.49–2.23	28	15.6	**1.80**	1.20–2.60
Printers	4	2.9	1.39	0.38–3.56	1	1.7	0.60	0.02–3.37	3	2.7	1.11	0.23–3.24	15	8.7	1.72	0.96–2.83	23	16.1	1.43	0.91–2.15
Chemical process workers	2	2.5	0.81	0.10–2.91	4	2.6	1.53	0.42–3.91	5	5.8	0.87	0.28–2.03	14	13.4	1.04	0.57–1.75	25	24.3	1.03	0.67–1.52
Food workers	9	8.2	1.09	0.50–2.08	3	1.9	1.61	0.33–4.70	8	6.6	1.22	0.53–2.40	17	13.3	1.28	0.75–2.05	37	30.2	1.22	0.86–1.69
Glass makers etc	6	4.8	1.26	0.46–2.74	4	2.8	1.43	0.39–3.65	10	3.3	**3.06**	1.47–5.64	13	14.7	0.89	0.47–1.51	33	25.6	1.29	0.89–1.81
Packers	8	4.2	1.92	0.83–3.78	6	5.5	1.09	0.40–2.37	8	10.0	0.80	0.35–1.58	26	27.0	0.96	0.63–1.41	48	46.9	1.02	0.76–1.36
Engine operators	11	4.4	**2.50**	1.25–4.47	10	7.9	1.26	0.61–2.33	4	5.9	0.68	0.19–1.75	24	22.5	1.07	0.68–1.59	49	41.0	1.20	0.88–1.58
Public safety workers	3	4.0	0.75	0.16–2.20	4	4.4	0.92	0.25–2.34	6	4.1	1.46	0.54–3.17	11	13.2	0.83	0.42–1.49	24	25.9	0.93	0.59–1.38
Cooks and stewards	0	0.3	0.00	0.00–10.59	1	0.3	2.99	0.08–16.68	4	2.0	2.05	0.56–5.25	7	2.6	**2.68**	1.08–5.53	12	5.4	**2.24**	1.16–3.91
Waiters	5	0.7	**6.81**	2.21–15.88	0	0.3	0.00	0.00–11.08	4	0.8	**4.79**	1.30–12.25	3	1.3	0.00	0.46–6.59	12	3.2	**3.69**	1.91–6.45
Building caretakers	2	4.2	0.48	0.06–1.72	7	3.6	1.96	0.79–4.04	1	2.2	0.46	0.01–2.54	13	10.9	1.19	0.63–2.04	23	20.9	1.10	0.70–1.65
Hairdressers	2	1.19	1.69	0.20–6.09	0	0.1	0.00	0.00–46.17	2	0	0.64	0.00–5.79	6	2.6	2.33	0.85–5.07	8	4.5	1.78	0.77–3.51
Military personnel	0	2.2	0.00	0.00–1.67	1	1.6	0.63	0.02–3.53	2	3.5	0.57	0.07–2.05	4	7.5	0.53	0.15–1.37	7	14.8	**0.47**	0.19–0.97
Other economically active	35	30.8	1.14	0.79–1.58	13	16.1	0.81	0.43–1.39	32	25.5	1.25	0.86–1.77	70	69.5	1.01	0.79–1.27	154	143.4	1.07	0.91–1.26
Economically inactive	19	12.5	1.52	0.91–2.37	17	21.9	0.78	0.45–1.24	8	10.0	0.80	0.34–1.57	56	54.5	1.03	0.78–1.33	100	99.6	1.00	0.83–1.22
All categories	312		1.00	Ref	263		1.00	Ref	358		1.00	Ref	1037		1.00	Ref	1980		1.00	Ref

Statistically significant SIR values are bolded

*CI* confidence interval

**Table 2:** Observed (Obs) and expected (Exp) numbers of cancer of the nasopharynx and standardised incidence ratios (SIR) among women, by country and occupation. Only occupational categories with ≥5 cases (Nordic total) are shown. Country-specific numbers for Iceland not shown

	Denmark				Finland				Norway				Sweden				Nordic total			
Occupational category	Obs	Exp	SIR	95% CI	Obs	Exp	SIR	95% CI	Obs	Exp	SIR	95% CI	Obs	Exp	SIR	95% CI	Obs	Exp	SIR	95% CI
Health workers	11	6.2	1.79	0.89–3.20	1	6.8	0.15	0.00–0.82	5	5.0	0.99	0.32–2.32	20	19.6	1.02	0.62–1.58	37	38.1	0.97	0.68–1.34
Teachers	3	3.3	0.92	0.19–2.70	4	4.1	0.97	0.26–2.49	1	3.3	0.31	0.01–1.71	8	13.1	0.61	0.26–1.20	16	24.1	0.67	0.38–1.08
Religous workers etc	0	0.4	0.00	0.00–8.44	3	2.2	1.35	0.28–3.93	0	0.8	0.00	0.00–4.72	2	4.3	0.47	0.06–1.69	5	7.8	0.64	0.21–1.50
Clerical workers	14	13.5	1.04	0.57–1.75	15	13.5	1.11	0.62–1.84	8	10.2	0.78	0.34–1.55	34	30.9	1.10	0.76–1.54	72	69.0	1.04	0.82–1.31
Sales agents	1	0.1	8.65	0.22–48.18	3	2.0	1.48	0.30–4.32	3	1.6	1.88	0.39–5.49	3	5.2	0.58	0.12–1.70	10	9.0	1.11	0.53–2.04
Shop workers	8	8.7	0.92	0.40–1.81	9	6.9	1.30	0.60–2.47	6	8.4	0.72	0.26–1.56	22	20.2	1.09	0.68–1.65	45	44.9	1.00	0.73–1.34
Farmers	7	6.1	1.15	0.46–2.37	6	4.7	1.29	0.47–2.80	5	5.6	0.89	0.29–2.07	2	2.3	0.87	0.11–3.15	20	19.1	1.05	0.64–1.61
Gardeners	2	0.8	2.52	0.31–9.12	11	12.5	0.88	0.44–1.58	4	3.3	1.22	0.33–3.13	6	5.5	1.09	0.40–2.38	23	22.0	1.04	0.66–1.57
Textile workers	2	3.0	0.67	0.08–2.41	4	4.8	0.84	0.23–2.15	4	3.5	1.16	0.31–2.96	9	12.8	0.70	0.32–1.34	19	24.3	0.78	0.47–1.22
Postal workers	1	0.5	2.16	0.05–12.05	3	2.1	1.42	0.29–4.15	1	2.0	0.49	0.01–2.74	6	5.6	1.08	0.40–2.35	12	10.4	1.15	0.60–2.01
Mechanics	1	0.6	1.75	0.04–9.73	0	0.6	0.00	0.00–5.84	0	0.3	0.00	0.00–12.95	4	2.4	1.67	0.46–4.28	5	3.9	1.29	0.42–3.00
Food workers	2	2.2	0.91	0.11–3.27	1	1.6	0.63	0.02–3.50	2	1.6	1.24	0.15–4.49	3	2.7	1.12	0.23–3.28	10	8.8	1.13	0.54–2.09
Glass makers etc	2	0.6	3.59	0.43–12.97	0	1.1	0.00	0.00–3.26	2	0.5	3.75	0.45–13.56	4	2.2	1.83	0.50–4.69	8	4.4	1.81	0.78–3.56
Packers	0	0.2	0.00	0.00–23.66	2	2.0	1.01	0.12–3.64	0	1.2	0.00	0.00–2.96	3	3.4	0.89	0.18–2.61	5	6.8	0.73	0.24–1.71
Cooks and stewards	0	0.0	0.00	0.00–664.38	5	2.3	2.15	0.70–5.01	4	1.9	2.14	0.58–5.47	7	5.5	1.27	0.51–2.61	16	10.0	1.60	0.92–2.60
Domestic assistants	2	3.3	0.60	0.07–2.16	1	2.6	0.39	0.01–2.17	1	4.9	0.21	0.01–1.15	14	14.1	0.99	0.54–1.66	18	25.0	0.72	0.43–1.14
Waiters	2	0.6	3.33	0.40–12.04	1	1.9	0.53	0.01–2.93	4	1.9	2.09	0.57–5.35	8	5.2	1.55	0.67–3.05	15	9.6	1.56	0.87–2.57
Building caretakers	8	9.7	0.83	0.36–1.63	15	7.9	**1.90**	1.07–3.14	11	7.8	1.40	0.70–2.51	10	14.1	0.71	0.34–1.30	44	40.0	1.10	0.80–1.48
Launderers	2	1.1	1.86	0.22–6.71	0	0.6	0.00	0.00–6.03	4	0.7	**5.38**	1.47–13.79	4	2.9	1.39	0.38–3.55	11	5.4	**2.04**	1.02–3.65
Other economically active	9	8.4	1.07	0.49–2.04	13	11.0	1.19	0.63–2.03	7	7.9	0.88	0.35–1.82	14	20.5	0.68	0.37–1.14	43	48.9	0.88	0.64–1.18
Economically inactive	60	67.8	0.89	0.68–1.14	50	55.8	0.90	0.66–1.18	67	66.5	1.01	0.78–1.28	304	294.6	1.03	0.92–1.15	484	486.5	0.99	0.91–1.09
All categories	137		1.00	Ref	147		1.00	Ref	139		1.00	Ref	487		1.00	Ref	918		1.00	Ref

Statistically significant SIR values are bolded

*CI* confidence interval

The SIR was significantly elevated among male cooks and stewards in the entire Nordic cohort (SIR 2.24, 95% CI 1.16–3.91) and in the Swedish cohort (SIR 2.68, 95% CI 1.08–5.53). The incidence of NPC was consistently elevated among male bricklayers in all countries. Finnish male administrators and sales agents had significantly elevated SIRs, but similar findings were not observed in other Nordic countries.

Among females, launderers had the highest and significantly elevated SIR in the entire cohort (SIR 2.04, 95% CI 1.02–3.65) and separately in the Norwegian cohort (SIR 5.38, 95% CI 1.47–13.79).

Significantly decreased incidence of NPC was found among Nordic male farmers (SIR 0.79, 95% CI 0.68–0.92) and male textile workers (SIR 0.49, 95% CI 0.22–0.93). The SIR for farmers was especially low in Finland and Denmark (SIR 0.58, 95% CI 0.36–0.88 and 0.65, 95% CI 0.42–0.95, respectively).

Male fishermen in Norway had a significantly elevated SIR (1.64, 95% CI 1.03–2.49, Table 1), and SIRs for both fishermen and seamen were consistently elevated in all countries except for Swedish fishermen, who had a low SIR.

## Discussion

This study provides new evidence on occupational risk variation of NPC in the Nordic countries. Norwegian, Finnish and Swedish fishermen had elevated SIRs of NPC in the present study, which to our knowledge, is a new finding. In addition, the SIRs for seamen were consistently elevated. Salted fish is a confirmed risk factor for NPC and commonly used in Southern Asia [27], but we have no evidence whether fishermen in the three Nordic countries consume more salted fish than the reference population. Fishermen might have been exposed to off-gas from fermented fish, especially in former times when the storage tanks on fishing boards were emptied manually.

Cooks and stewards showed elevated SIRs in the present study. The elevated risk for lung cancer among cooks has been previously related to indoor air pollution, which may also be one link between cooks and NPC [28]. In addition, wood combustion has been found to be a potential risk factor for NPC and a potential exposure agent for cooks [29].

In some previous studies, frequent exposure to cotton dust has been associated with elevated risk of NPC [12, 21, 30]. We did not find an elevated SIR among female textile workers, and there was a statistically significant decreased SIR for male textile workers. Thus, this study does not support such evidence.

We found that female launderers had statistically significantly higher risk of NPC than the population on average. In drycleaning, tetrachloroethylene is the dominant solvent used throughout the world and in the Nordic countries [31].

The IARC classified formaldehyde and wood dust as occupational carcinogens in relation to NPC. In the present study, exposure to these agents is most likely among woodworkers, but we did not find elevated SIRs among them [32].

NPC incidence was significantly elevated among male waiters. Previously, Nordic waiters have been related to elevated incidence of lung cancer, oral cancer and pharyngeal cancer [33]. An increased risk of death from NPC was reported among waiters in China [34]. One explanation may be that waiters have been exposed to passive and active tobacco smoke, which has also been associated with NPC [4, 35]. Prior to smoking restrictions in bars and restaurants, waiters worked in a smoke-filled environment and had a higher proportion of active smokers than other workers [33]. Several studies have shown a modestly increased risk of NPC associated with tobacco smoking, and a meta-analysis indicated an approximately 60% increased risk for NPC among smokers [36, 37]. In Finland and Norway, smoke-free legislation in restaurants has shown effective results in the decrease of passive smoking exposure [35, 38] and improving lung function [39] among waiters. Therefore, it is expected that the excess incidence of NPC among restaurant staff will become smaller in the future.

Strengths of this study include the large cohorts with nationwide population-based data, long follow-up periods and high quality registry data [26]. A limitation of the present study was the missing stratification of NPCs by histological subtype, and the lack of information on factors not directly related to occupation. It has been demonstrated that the role of tobacco and alcohol use in the occupational risk variation in the risk for head neck cancer in the Nordic countries is large [40]. In addition, since the current data only reach to the end of the year 2005, they mainly reflect occupational exposures before the mid-1990s, considering the long latency in cancer process.

## Conclusions

The present study suggests that certain work-related exposures such as passive smoking, kitchen air pollution and solvents may be associated with NPC. In future, exposure-risk relations should be studied to understand more about causality and to assess effective prevention strategies against NPC.

## Data Availability

The tabulated datasets generated and/or analysed during the current study are available in the NOCCA repository, https://astra.cancer.fi/NOCCA/.

## Abbreviations

EBV: Epstein-Barr virus
CI: Confidence interval
HNSCC: Head and neck squamous cell carcinoma
HPV: Human papillomavirus
IARC: International Agency for Research on Cancer
ISCO: International Standard of Classification of Occupations
NOCCA: Nordic Occupational Cancer Study
NPC: Nasopharyngeal carcinoma
NYK: Nordic Occupational Classification
SIR: Standardized incidence ratio
